# Multicenter Noninferiority Evaluation of Hain GenoType MTBDR*plus* Version 2 and Nipro NTM+MDRTB Line Probe Assays for Detection of Rifampin and Isoniazid Resistance

**DOI:** 10.1128/JCM.00251-16

**Published:** 2016-05-23

**Authors:** Ruvandhi R. Nathavitharana, Doris Hillemann, Samuel G. Schumacher, Birte Schlueter, Nazir Ismail, Shaheed Vally Omar, Welile Sikhondze, Joshua Havumaki, Eloise Valli, Catharina Boehme, Claudia M. Denkinger

**Affiliations:** a Division of Infectious Diseases, Beth Israel Deaconess Medical Center, Boston, Massachusetts, USA; b National Reference Laboratory for Mycobacteria, Forschungszentrum Borstel, Borstel, Germany; c FIND, Geneva, Switzerland; d Centre for Tuberculosis, National Institute for Communicable Diseases, Johannesburg, South Africa; e National TB Control Program, Ministry of Health, Swaziland

## Abstract

Less than 30% of multidrug-resistant tuberculosis (MDR-TB) patients are currently diagnosed, due to laboratory constraints. Molecular diagnostics enable rapid and simplified diagnosis. Newer-version line probe assays have not been evaluated against the WHO-endorsed Hain GenoType MTBDR*plus* (referred to as Hain version 1 [V1]) for the rapid detection of rifampin (RIF) and isoniazid (INH) resistance. A two-phase noninferiority study was conducted in two supranational reference laboratories to allow head-to-head comparisons of two new tests, Hain Genotype MTBDR*plus* version 2 (referred to as Hain version 2 [V2]) and Nipro NTM+MDRTB detection kit 2 (referred to as Nipro), to Hain V1. In phase 1, the results for 379 test strains were compared to a composite reference standard that used phenotypic drug susceptibility testing (DST) and targeted sequencing. In phase 2, the results for 644 sputum samples were compared to a phenotypic DST reference standard alone. Using a challenging set of strains in phase 1, the values for sensitivity and specificity for Hain V1, Hain V2, and Nipro, respectively, were 90.3%/98.5%, 90.3%/98.5%, and 92.0%/98.5% for RIF resistance detection and 89.1%/99.4%, 89.1%/99.4%, and 89.6%/100.0% for INH resistance detection. Testing of sputa in phase 2 yielded values for sensitivity and specificity of 97.1%/97.1%, 98.2%/97.8%, and 96.5%/97.5% for RIF and 94.4%/96.4%, 95.4%/98.8%, and 94.9%/97.6% for INH. Overall, the rates of indeterminate results were low, but there was a higher rate of indeterminate results with Nipro than with Hain V1 and V2 in samples with low smear grades. Noninferiority of Hain V2 and Nipro to Hain V1 was demonstrated for RIF and INH resistance detection in isolates and sputum specimens. These results serve as evidence for WHO policy recommendations on the use of line probe assays, including the Hain V2 and Nipro assays, for MDR-TB detection.

## INTRODUCTION

Multidrug-resistant tuberculosis (MDR-TB) poses complex diagnostic challenges and is a major obstacle to TB control. Less than 30% of existing MDR-TB patients are currently being diagnosed, due to critical laboratory capacity constraints (18). Conventional drug susceptibility testing (DST) using solid or liquid culture methods takes several weeks (2, 3). Genotypic (molecular) methods for MDR-TB detection offer considerable advantages, including rapidity of diagnosis, potential for high throughput, and decreased laboratory biosafety requirements (4, 5). In 2008, WHO endorsed the use of line probe assays (LPAs) for the rapid detection of MDR-TB (6), beginning what might be considered to be the molecular revolution in TB diagnosis.

The WHO-endorsed LPA, GenoType MTBDR*plus* (subsequently referred to as Hain version 1 [V1]), includes *rpoB* probes to determine rifampin (RIF) resistance, along with *katG* and *inhA* probes to determine high-level and low-level isoniazid (INH) resistance, respectively (1). The pooled sensitivity and specificity estimates for RIF resistance were 98.1% and 98.7%, respectively, and the individual values were highly consistent among all patient subgroups and specimen types in a meta-analysis (7). The pooled sensitivity estimate for INH resistance was lower, at 84.3%, and the individual values were more varied, while the pooled specificity estimate was high, at 99.5%.

Hain introduced an updated version of the MTBDR*plus* LPA in 2011 (subsequently referred to as Hain version 2 [V2]), which has the same set of probes for wild-type and specific mutations as Hain V1. Also in 2011, Nipro Corporation completed the development of their LPA, NTM+MDRTB detection kit 2 (subsequently referred to as Nipro). This assay also enables the detection of resistance to RIF and INH and M. tuberculosis complex (MTC). The *rpoB*, *katG*, and *inhA* mutation probes are the same for the three assays, although there are some minor variations between the Nipro and Hain V1/V2 assays in the codon regions covered for the wild type. Initial publications evaluating these second-generation and new LPAs have demonstrated promising results (8–10). However, to date, the absence of head-to-head comparison data makes it difficult to judge whether the second-generation LPAs perform at least as well as the WHO-endorsed Hain V1.

The purpose of this study was to evaluate and directly compare the performance of the index tests, Hain V2 and Nipro, and the comparator test, the WHO-endorsed Hain V1, for the detection of RIF and INH resistance in MTC clinical isolates and sputum specimens.

## MATERIALS AND METHODS

### Study design and phases.

This prospective, multicenter, blinded, diagnostic accuracy study was designed to allow direct comparisons between the index tests (Hain V2 and Nipro) and the comparator test (WHO-approved Hain V1) by testing a set of samples with all three tests, as well as the reference standard. Phase 1 adopted a case-control design, in which each participating laboratory evaluated 200 strains from the Research and Training in Tropical Diseases (TDR) and Institute for Tropical Medicine (ITM) collections (see Appendix SA, Table SA1 in the supplemental material for details of strain selection). The sites were blinded to phenotypic and genotypic results for the strains.

Phase 2 adopted a cross-sectional design and evaluated 644 residual sputum samples from patients being evaluated for pulmonary TB who were at risk of MDR-TB based on the geographic regions from which they were sampled (see Appendix SA, Table SA2 in the supplemental material for details of sputum specimen selection). The residual sputum samples tested in South Africa were obtained from patients presenting locally, whereas the residual sputum samples tested in Germany originated from Azerbaijan and Moldova. Consecutive sputum specimens were collected over a 2-month period. Smear-positive and a small subset of smear-negative samples were tested (see Appendix SC, Table SC2).

### Index test and reference standard test procedures.

The index tests were performed in accordance with the manufacturer's instructions. The minimum reference standard for medium resistance detection was considered to be phenotypic DST by MIC testing on Lowenstein-Jensen medium or MGIT SIRE (streptomycin, INH, RIF, and ethambutol) using WHO standard concentrations.

Although phenotypic DST has hitherto been considered the “gold standard” for the determination of drug resistance, there is increasing evidence that strains found to be sensitive by phenotypic DST may carry mutations as determined by genotypic DST that can be clinically significant (11, 14). The optimal reference standard for resistance detection, therefore, was considered to be a composite reference standard of phenotypic DST and sequencing. Sanger sequencing (see Appendix SB in the supplemental material) of specific DNA fragments was performed on all discrepant strains and a subset of nondiscrepant strains (reclassified strains are detailed in Appendix SD), and the composite reference standard constructed was blinded to LPA results. Sequencing was only performed on the test strains in phase 1 and was done at the National Reference Laboratory in Germany. For phase 2, phenotypic DST alone was used as the reference standard.

For phase 1 (testing on strains), an additional analysis was performed to detail the reasons for false-positive and false-negative LPA results. Incorrect LPA results were classified as “failure of the design” if the error was due to the test design (i.e., because the resistance-conferring mutation was not covered by the assay). Incorrect results were classified as “failure of the assay” if the error occurred because the test did not detect a mutation it should actually have detected.

### Study procedures.

For phase 1, the strains were supplied to the study sites as DNA suspensions. These were prepared by transferring grown colonies into Tris-EDTA buffer (10 nM Tris-HCl, 1 nM EDTA [pH 8.0]) and boiling for 5 min. Each tube contained 200 to 400 μl of heat-inactivated bacterial DNA suspension, and 5 μl was required to run each assay.

For phase 2, leftover sediment of selected decontaminated sputum specimens was used. The sputa had originally been processed by the conventional *N*-acetyl-l-cysteine-NaOH method (final NaOH concentration, 1%). The concentrated sediment was suspended in 1.6 to 2.0 ml sterile phosphate buffer (pH 7.0), tested by culture and DST, and then stored at −20°C or −80°C. The leftover sediments of selected specimens were thawed and used for testing by the index tests as follows: 500 μl was used for inactivation and DNA isolation (using an ultrasonic bath) and tested with Hain V1 and Nipro, while 500 μl was used for inactivation and DNA extraction by the GenoLyse (Hain Lifescience) method and tested with the Hain V2. After DNA extraction, PCR is performed, followed by immobilization of the amplification products to specifically prepared membranes. A subsequent enzyme-driven color reaction shows the binding of amplification products to their corresponding sequences on the membrane, resulting in a band pattern. Necessary components for PCR and hybridization are included in the kits. There are differences in the PCR programs, primer design, probe coverage, hybridization temperatures, and devices, whereas the genes investigated (i.e., *katG*, *inhA*, and *rpoB*) are identical.

As specified by the manufacturers' instructions, test results were regarded as “invalid” if the conjugate/color control or the amplification control was negative. Test results were defined as “indeterminate” if the test results were valid but readers were unable to draw conclusions on RIF/INH resistance based on the visible banding pattern. If an LPA test was repeated based on an initially invalid or indeterminate result, the repeat results were used for the analyses of test accuracy. Invalid and indeterminate results on initial and repeat testing were reported separately and excluded from analyses of diagnostic accuracy. Samples for which reference standard results were missing (e.g., due to contamination) were excluded from the analysis.

### Study outcomes and analyses of outcome data.

The primary study outcomes were sensitivity and specificity for detection of RIF and INH resistance. Sensitivity and specificity were based on samples with valid results on both the index test (either Hain V2 or Nipro) and the comparator test (Hain V1) for each comparison. The key endpoint was the difference in accuracy for the detection of RIF and INH resistance between the index tests (Hain V2 and Nipro) and the comparator (Hain V1) in reference to the noninferiority margins (see Table 1). To evaluate noninferiority of the index tests, the 95% confidence interval (CI) of this difference was calculated. Noninferiority was demonstrated if the lower limit of this 95% confidence interval did not lie below the noninferiority margin (12, 13). Noninferiority was assessed based on pooled estimates from all sites. The secondary study outcomes were the rates of indeterminate results and the ease of use (see Appendix SF in the supplemental material).

**TABLE 1:** Noninferiority margins used to compare Hain V2 and Nipro to Hain V1

Reference standard used, study phase	Noninferiority margin (%) for:			
	RIF		INH	
	Sensitivity	Specificity	Sensitivity	Specificity
Composite, 1	−3	−2	−10	−5
Phenotypic, 2	−5	−4	−12	−7

For phase 2, sequencing was not performed. Given that this may lead to underestimation of the accuracy of the index tests (i.e., when LPA detects disputed mutations in strains that are phenotypically sensitive), the noninferiority margins for resistance detection were widened by 2% for each comparison.

Statistical analysis was performed using STATA version 13 and R. Confidence intervals or proportions were computed with the exact binomial method (Clopper-Pearson confidence intervals). Confidence intervals for differences in proportions (to assess noninferiority) were computed using Tango's confidence interval, which accounts for the paired nature of the data.

### Ethics.

Each study site obtained local Institutional Review Board (IRB) approval. No foreseen risks were anticipated with this study, and no patient identifying information was collected.

## RESULTS

### Phase 1 evaluation: testing of strains. (i) Comparative accuracy on strains. *(a)* Hain V2 versus Hain V1.

The levels of accuracy of both Hain V1 and Hain V2 were high, and the two tests had identical point estimates for RIF, INH, and MDR sensitivity and specificity. Compared to the composite reference standard, both Hain V1 and V2 gave the following results: RIF sensitivity, 90.3%; RIF specificity, 98.5%; INH sensitivity, 89.1%; INH specificity, 99.4%; MDR sensitivity, 83.9%; and MDR specificity, 99.1%. Using the phenotypic DST reference standard improved the sensitivity slightly further while reducing the specificity slightly for RIF, INH, and MDR.

**FIG 1:**
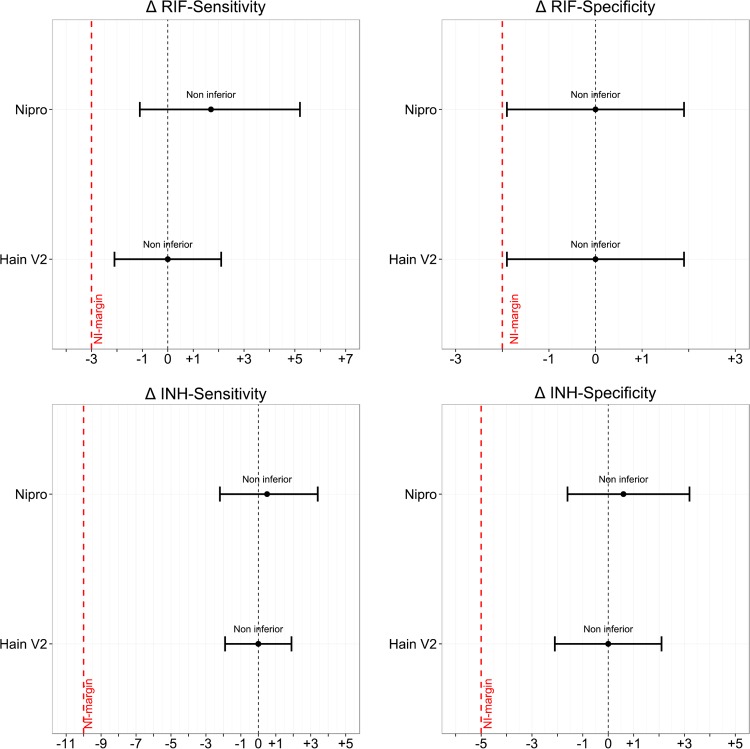
Comparative accuracy of Hain V2 and Nipro versus Hain V1 for strains using the composite reference standard. The differences in accuracy parameters (Δ = Hain V2/Nipro – Hain V1) are displayed as horizontal lines, with points representing the point estimate and whiskers representing the upper and lower limits of the 95% CIs. The black vertical dotted line indicates zero difference in the levels of sensitivity/specificity, and the red vertical broken line indicates the noninferiority margin. Noninferiority is demonstrated for a given comparison if the lower limit of the 95% CI does not cross the red broken line (noninferiority margin).

Noninferiority was demonstrated for all parameters for Hain V2 in comparison to Hain V1, with the lower levels of the 95% CIs of the differences in sensitivity and specificity being above the limit set by the noninferiority margins specified (Table 2 and Fig. 1).

**TABLE 2:** Accuracy of Hain V2 versus Hain V1 when testing strains

Drug	Parameter	Estimate [% (95% CI)] [no. of strains for which results were in agreement/total no. of strains tested] using indicated reference standard for indicated test				Difference (Hain V2 − Hain V1) [% (95% CI)]	NI margin (%)^*a*^
		Phenotypic DST		Composite of phenotypic DST and sequencing			
		Hain V1	Hain V2	Hain V1	Hain V2		
RIF	Sensitivity	91.3 (86.0, 95.0) [157/172]	91.3 (86.0, 95.0) [157/172]	90.3 (84.9, 94.2) [158/175]	90.3 (84.9, 94.2) [158/175]	0.0 (−2.1, 2.1)	−3
	Specificity	98.0 (95.0, 99.5) [198/202]	98.0 (95.0, 99.5) [198/202]	98.5 (95.7, 99.7) [196/199]	98.5 (95.7, 99.7) [196/199]	0.0 (−1.9, 1.9)	−2
INH	Sensitivity	89.4 (84.3, 93.3) [178/199]	89.4 (84.3, 93.3) [178/199]	89.1 (83.9, 93.0) [179/201]	89.1 (83.9, 93.0) [179/201]	0.0 (−1.9, 1.9)	−10
	Specificity	98.9 (96.0, 99.9) [175/177]	98.9 (96.0, 99.9) [175/177]	99.4 (96.9, 100.0) [174/175]	99.4 (96.9, 100.0) [174/175]	0.0 (−2.1, 2.1)	−5
MDR	Sensitivity	84.3 (77.6, 89.7) [129/153]	84.3 (77.6, 89.7) [129/153]	83.9 (77.1, 89.3) [130/155]	83.9 (77.1, 89.3) [130/155]	0.0 (−2.4, 2.4)	NA
	Specificity	98.6 (96.1, 99.7) [218/221]	98.6 (96.1, 99.7) [218/221]	99.1 (96.7, 99.9) [217/219]	99.1 (96.7, 99.9) [217/219]	0.0 (−1.7, 1.7)	NA

a The noninferiority (NI) margin is presented only for the composite reference standard section because this was the reference standard selected for the analysis.

### *(b)* Nipro versus Hain V1.

The levels of accuracy of both Hain V1 and Nipro were high and overall very similar, irrespective of the reference standard used. Compared to the composite reference standard, the accuracy of Nipro was identical to or slightly better than that of Hain V1, as follows: RIF sensitivity of 92.0% versus 90.3%, respectively, identical RIF specificities of 98.5%, INH sensitivity of 89.6% versus 89.1%, INH specificity of 100% versus 99.4%, MDR sensitivity of 85.2% versus 83.9%, and identical MDR specificities of 99.1%. Using the phenotypic DST reference standard improved the sensitivity slightly further while reducing the specificity slightly for RIF, INH, and MDR.

Noninferiority was demonstrated for all parameters for Nipro in comparison to Hain V1, with the lower levels of the 95% CIs of the differences in sensitivity and specificity being above the limit set by the noninferiority margins specified (Table 3 and Fig. 1).

**TABLE 3:** Accuracy of Nipro versus Hain V1 on strains

Drug	Parameter	Estimate [% (95% CI)] [no. of strains for which results were in agreement/total no. of strains tested] using indicated reference standard for indicated test				Difference (Nipro − Hain V1) [% (95% CI)]	NI margin (%)^*a*^
		Phenotypic DST		Composite of phenotypic DST and sequencing			
		Hain V1	Nipro	Hain V1	Nipro		
RIF	Sensitivity	91.3 (86.0, 95.0) [157/172]	92.4 (87.4, 95.9) [159/172]	90.3 (84.9, 94.2) [158/175]	92.0 (86.9, 95.6) [161/175]	+1.7 (−1.1, 5.2)	−3
	Specificity	98.0 (95.0, 99.5) [198/202]	97.5 (94.3, 99.2) [197/202]	98.5 (95.7, 99.7) [196/199]	98.5 (95.7, 99.7) [196/199]	0.0 (−1.9, 1.9)	−2
INH	Sensitivity	89.4 (84.3, 93.3) [178/199]	89.9 (84.9, 93.8) [179/199]	89.1 (83.9, 93.0) [179/201]	89.6 (84.5, 93.4) [180/201]	+0.5 (−2.2, 3.4)	−10
	Specificity	98.9 (96.0, 99.9) [175/177]	99.4 (96.9,100.0) [176/177]	99.4 (96.9, 100.0) [174/175]	100.0 (97.9, 100.0) [175/175]	+0.6 (−1.6, 3.2)	−5
MDR	Sensitivity	84.3 (77.6, 89.7) [129/153]	85.0 (78.3, 90.2) [130/153]	83.9 (77.1, 89.3) [130/155]	85.2 (78.6, 90.4) [132/155]	+1.3 (−2.3, 5.3)	NA
	Specificity	98.6 (96.1, 99.7) [218/221]	98.2 (95.4, 99.5) [217/221]	99.1 (96.7, 99.9) [217/219]	99.1 (96.7, 99.9) [217/219]	0.0 (−1.7, 1.7)	NA

a The noninferiority (NI) margin is presented only for the composite reference standard section because this was the reference standard selected for the analysis.

### (ii) Rates of invalid controls and indeterminate test results.

Three hundred seventy-nine MTC strains were tested with all three LPAs (189 in Germany and 190 in South Africa; see Appendix SA in the supplemental material for strain composition). There was one invalid control result for Nipro and none for Hain V1 or Hain V2. Indeterminate results were rare (<1%) for Hain V1 and Hain V2 (see Appendix SC, Table SC1). The rates of indeterminate results for Nipro were also low at the South African site (1.1% and 0.5% for RIF and INH, respectively) but were higher at the German site (5.3% and 4.3% for RIF and INH, respectively) on initial testing. Retesting strains that initially tested indeterminate using Nipro in Germany resolved most indeterminate results. Repeat testing was not performed in South Africa.

### Phase 2 evaluation: testing of sputa. (i) Comparative accuracy on sputum specimens. *(a)* Hain V2 versus Hain V1.

The levels of accuracy of both Hain V2 and Hain V1 for direct testing on sputum specimens were high and overall similar. Hain V2 performed slightly better than Hain V1 across all four accuracy parameters, as follows: RIF sensitivity of 98.2% versus 97.1%, respectively, RIF specificity of 97.8% versus 97.1%, INH sensitivity of 95.4% versus 94.4%, and INH specificity of 98.8% versus 96.4%. The MDR sensitivity was 96.7% versus 94.7%, and the MDR specificity was 98.3% versus 96.9%.

**FIG 2:**
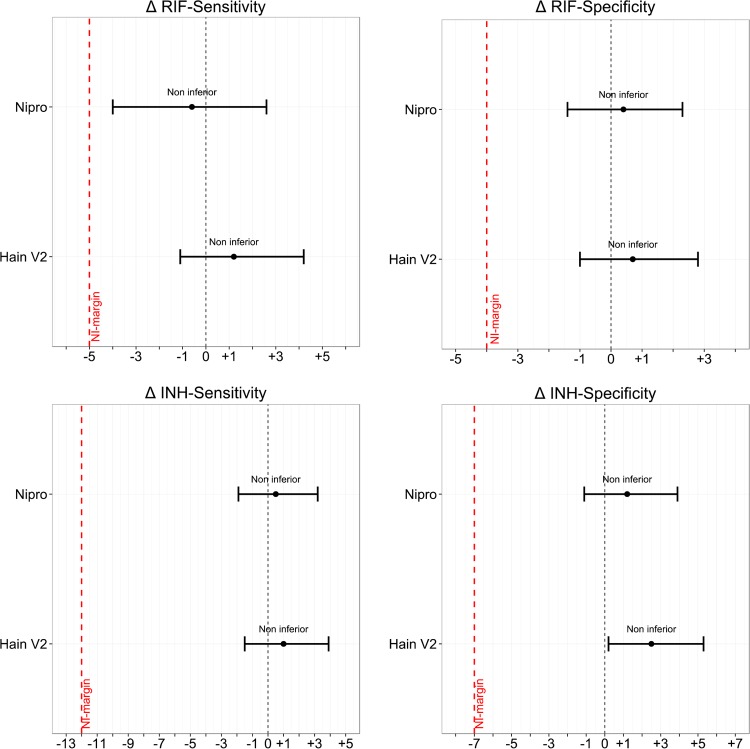
Comparative accuracy of Hain V2 and Nipro versus Hain V1 for sputa using the phenotypic reference standard. The differences in accuracy parameters (Δ = Hain V2/Nipro – Hain V1) are displayed as horizontal lines, with points representing the point estimate and whiskers representing the upper and lower limits of the 95% CIs. The black vertical dotted line indicates zero difference in the levels of sensitivity/specificity, and the red vertical broken line indicates the noninferiority margin. Noninferiority is demonstrated for a given comparison if the lower limit of the 95% CI does not cross the red broken line (noninferiority margin).

Noninferiority was demonstrated for all parameters for Hain V2 in comparison to Hain V1, with the lower levels of the 95% CIs of the differences in sensitivity and specificity being above the limit set by the noninferiority margins specified. The differences in sensitivity and specificity with 95% CIs in relation to the noninferiority margin are shown (Table 4 and Fig. 2).

**TABLE 4:** Comparative accuracy of Hain V2 or Nipro versus Hain V1 on sputa, using phenotypic reference standard

Drug	Parameter	Estimate [% (95% CI)] [no. of strains for which results were in agreement/total no. of strains tested] by:		Difference (Hain V2 − Hain V1) [% (95% CI)]	NI margin (%)	Estimate [% (95% CI)] [no. of strains for which results were in agreement/total no. of strains tested] by:		Difference (Nipro − Hain V1) [% (95% CI)]	NI margin (%)
		Hain V1	Hain V2			Hain V1	Nipro		
RIF	Sensitivity	97.1 (93.3, 99.0) [166/171]	98.2 (95.0, 99.6) [168/171]	+1.2 (−1.1, 4.2)	−5	97.1 (93.3, 99.0) [166/171]	96.5 (92.5, 98.7) [165/171]	−0.6 (−4.0, 2.6)	−5
	Specificity	97.1 (94.3, 98.7) [267/275]	97.8 (95.3, 99.2) [269/275]	+0.7 (−1.0, 2.8)	−4	97.1 (94.3, 98.7) [267/275]	97.5 (94.8, 99.0) [268/275]	+0.4 (−1.4, 2.3)	−4
INH	Sensitivity	94.4 (90.2, 97.2) [186/197]	95.4 (91.5, 97.9) [188/197]	+1.0 (−1.5, 3.9)	−12	94.4 (90.2, 97.2) [186/197]	94.9 (90.9, 97.5) [187/197]	+0.5 (−1.9, 3.2)	−12
	Specificity	96.4 (93.2, 98.3) [240/249]	98.8 (96.5, 99.8) [246/249]	+2.4 (0.2, 5.3)	−7	96.4 (93.2, 98.3) [240/249]	97.6 (94.8, 99.1) [243/249]	+1.2 (−1.1, 3.9)	−7
MDR	Sensitivity	94.7 (89.9, 97.7) [144/152]	96.7 (92.5, 98.9) [147/152]	+2.0 (−0.5, 5.6)	NA	94.7 (89.9, 97.7) [144/152]	94.7 (89.9, 97.7) [144/152]	0.0 (−3.5, 3.5)	NA
	Specificity	96.9 (94.1, 98.6) [278/287]	98.3 (96.0, 99.4) [282/287]	+1.4 (0.05, 3.5)	NA	96.9 (94.1, 98.6) [278/287]	97.6 (95.0, 99.0) [280/287]	+0.7 (−0.6, 2.5)	NA

### *(b)* Nipro versus Hain V1.

The levels of accuracy of both Nipro and Hain V1 for direct testing on sputum specimens were high and overall similar. Nipro performed slightly better than Hain V1 for all accuracy parameters apart from RIF sensitivity and MDR sensitivity. Nipro performed slightly worse than Hain V1 for RIF detection, with a sensitivity of 96.5% versus 97.1%, respectively, although its specificity for RIF detection was slightly higher, at 97.5% versus 97.1%. INH detection for Nipro compared to Hain V1 was slightly higher, with sensitivity of 94.9% versus 94.4% and specificity of 97.6% versus 96.4%. The levels of MDR sensitivity were identical at 94.7%, and MDR specificity was 97.6% versus 96.9%.

Noninferiority was demonstrated for all parameters for Nipro in comparison to Hain V1, with the lower levels of the 95% CIs of the differences in sensitivity and specificity being above the limits set by the specified noninferiority margins (Table 4 and Fig. 2).

### (ii) Rates of invalid controls and indeterminate test results.

In phase 2, 270 and 229 MTC culture-positive sputum specimens were tested with all three LPAs in Germany and South Africa, respectively. There were no invalid control results for any of the tests at either the German or South African site. The rates of indeterminate results were below 4% for Hain V1 and below 2% for Hain V2 but around 6% for Nipro on initial testing (see Appendix SC in the supplemental material).

Less than half (37%) of all indeterminate results were due to MTC not being detected at all by the LPAs (and thus, the resistance status remaining unknown, i.e., indeterminate); the other samples testing indeterminate were identified as MTC, but the banding pattern for RIF and/or INH did not allow the determination of drug resistance status. The rates of indeterminate results of Hain V2 seemed largely unaffected by the smear grade; this was similar for Hain V1, although the rates of indeterminate results did increase somewhat in scanty samples. Nipro had rates of indeterminate results similar to those of the Hain assays for samples with smear grades of 2+ and 3+ but higher rates of indeterminate results in samples with smear grades of 1+ and scanty. All three LPAs had relatively high rates of indeterminate results in smear-negative/culture-positive samples, although Hain V2 had markedly lower rates than Hain V1 and Nipro had much higher rates than both Hain V1 and Hain V2. Repeat testing was done on some of the samples, and the results are presented in Appendix SC, Table SC1 in the supplemental material.

## DISCUSSION

In this first head-to-head comparison of three LPAs, Hain V2 and Nipro were both noninferior to the WHO-endorsed Hain V1 for RIF and INH resistance detection when used indirectly on culture isolates and directly on decontaminated sputum samples. All three LPAs showed high levels of accuracy, with only minor variations between them.

Although the differences between the three tests for the detection of RIF and INH resistance were minimal, there were some notable differences in the rates of indeterminate results on strains and, in particular, on sputum samples with lower smear grades. Overall, the rates of indeterminate results were low, which may be because testing was performed at the national reference laboratory level and because of the exclusion of samples containing blood. The Hain V2 assay appeared even more robust than the Hain V1 for lower MTC concentrations. The Nipro assay showed higher rates of indeterminate results at smear grades of 1+ and scanty, suggesting that either the extraction or amplification steps were not as efficient as with the Hain assay. Indeterminate results may be resolved with repeat testing, but this implies the need to use additional assays and labor and may lead to delays in time to diagnosis. This might be outweighed by a lower cost per test of the Nipro assay. Currently, the cost for the Nipro assay is still being defined. The FIND-negotiated cost for the Hain LPAs is ∼$8.

Compared to analyses based on the phenotypic reference standard, analyses based on the composite reference standard tended to yield somewhat lower levels of sensitivity and somewhat higher levels of specificity. This fits expectations, since the addition of genotypic data allows reclassification of samples testing sensitive phenotypically but resistant genotypically, but not the other way around. Three of four strains classified as RIF sensitive by phenotypic DST but RIF resistant by composite DST carried the L533P mutation (change of leucine to proline at residue 533) (see Table SD1 in the supplemental material), one of which was classified as resistant by all 3 assays and one of which was only identified as resistant by Nipro. The L533P mutation has been demonstrated to be associated with worse clinical outcomes, and so, detection of this mutation by LPA offers potential clinical advantages over phenotypic DST (14). Both of the strains classified as INH sensitive on phenotypic DST but INH resistant by composite DST carried the C15T *inhA* mutation, which is associated with low-level INH resistance. *inhA* mutations also confer resistance to ethionamide, which is commonly used in second-line regimens (15, 16). Knowledge of specific mutations as reported by LPAs allows clinicians to further adjust treatment regimens accordingly.

We also noted that the estimates for assay sensitivity were higher for sputa (phase 2) than for strains (phase 1). This reflects the fact that a wide range of strains and resistance-conferring mutations were chosen to challenge the assays in phase 1, whereas in phase 2, the distribution of strains and mutations is likely more representative of routine clinical samples and more heavily weighted toward common mutations, which the assays detected with high accuracy. Accordingly, the test performance estimates from phase 2 were overall in accordance with previous reports in the literature where accuracy was estimated by testing routine clinical samples (9, 10, 17). The reasons for incorrect LPA results for phase 1 (in which sequencing was available) are divided into failure of the assay and of the design and are described in more detail in Appendix SE in the supplemental material.

This study had several important strengths. First, the same set of samples was tested with all three LPAs, allowing direct comparison of their performance, in contrast to other studies in which difference in performance may have been confounded when different strains were tested. Second, the two phases enabled the assays to be challenged with a wide array of strains and mutations (phase 1) and to be tested on routine sputa representing a real-life setting (phase 2). The sputum samples additionally included the full spectrum of smear grades, allowing the evaluation of representative overall rates of indeterminate results, as well as how rates vary across different concentrations of MTC. Third, the use of a composite reference standard that included genotypic data during phase 1 minimized bias and allowed for additional insights on test performance.

This study also had some limitations. Sequencing was not available during phase 2, and this could have led to some degree of misclassification of the true resistance status. However, we were able to inspect such an effect during phase 1, which suggested that this effect is minor. The Hain V1 assay had been used at the study sites prior to the study, potentially putting the Nipro assay at a disadvantage. Nonetheless, both sites were reference laboratories experienced in carrying out complex tests, and the test developers and site staff deemed that the training received was sufficient. Not all samples with indeterminate results were retested, thus limiting our ability to estimate the success rate upon retesting.

In summary, this study demonstrated that Hain V2 and Nipro were both noninferior to the WHO-endorsed Hain V1 for the detection of RIF and INH resistance when used on culture isolates and directly on decontaminated sputum samples. These data have been provided as evidence to support WHO policy recommendations on the use of LPAs, including the Hain V2 and Nipro assays, for rapid detection of MDR-TB in smear-positive samples.

## Supplementary Material

Supplemental material
