# Supplementary material for: Multicenter Noninferiority Evaluation of Hain GenoType MTBDRplus Version 2 and Nipro NTM+MDRTB Line Probe Assays for Detection of Rifampin and Isoniazid Resistance
Source: J Clin Microbiol. 2016 May 23;54(6):1624–30. doi: 10.1128/JCM.00251-16 (PMC4879293; doi:10.1128/JCM.00251-16)
Supplement: Supplemental material [file supp_54_6_1624__index.html]

Multicenter Noninferiority Evaluation of Hain GenoType MTBDRplus Version 2 and Nipro NTM+MDRTB Line Probe Assays for Detection of Rifampin and Isoniazid Resistance — Supplemental material 

# Multicenter Noninferiority Evaluation of Hain GenoType MTBDR*plus* Version 2 and Nipro NTM+MDRTB Line Probe Assays for Detection of Rifampin and Isoniazid Resistance

## Supplemental material

- Supplemental file 1 -

  Appendix SA (Baseline strain composition); Tables SA1 (Baseline strain composition for phase 1) and SA2 (Baseline composition of clinical isolates in phase 2); Appendices SB (Details of sequencing) and SC (Characterization of indeterminate results); Tables SC1 (Phase 1 indeterminate results on initial and repeat testing of strains) and SC2 (Phase 2 indeterminate results for RIF and INH on initial testing); Appendix SD (Details of reclassified strains according to sequencing); Tables SD1 (Reclassified strains according to sequencing for determination of RIF resistance) and SD2 (Reclassified strains according to sequencing for determination of INH resistance); Appendix SE (Analysis of reasons for test failure); Tables SE1 (Failure by design for RIF resistance detection), SE2 (Failure by assay for RIF resistance detection), SE3 (Failure by design for INH resistance detection), and SE4 (Failure by assay for INH resistance detection); and Appendix SF (Ease-of-use questionnaire results)

  PDF, 344K
