# Supplementary material for: Multicenter Noninferiority Evaluation of Hain GenoType MTBDRplus Version 2 and Nipro NTM+MDRTB Line Probe Assays for Detection of Rifampin and Isoniazid Resistance
Source: J Clin Microbiol. 2016 May 23;54(6):1624–30. doi: 10.1128/JCM.00251-16 (PMC4879293; doi:10.1128/JCM.00251-16)
Supplement: Supplemental material [file JCM.00251-16_zjm999095009so1.pdf]

Appendices

Appendix A: Baseline strain composition

Table A1. Baseline strain composition for Phase 1.

	Germany	South Africa	TOTAL
**RIF-R**	89	85	174
**INH-R**	102	99	201
**MDR**	81	74	155
**Total MTB strains**	**189**	**190**	**379**

Phase 1 strain selection

230 strains were available from the WHO-TDR-TB Strain bank (Vincent et al. 2012) to include a wide range of globally observed RIF and INH resistance mutations. An addition 20% RIF sensitive strains were included. 50 common MTB strains were sent to both sites, while the remaining 180 strains were divided between the two sites, ensuring a similar distribution of mutants and wild type for *rpoB*, *katG* and *inhA*. To reach the total number of 200 strains per site, 60 additional strains from the ITM's research collection were added per site, ensuring a similar distribution of resistance patterns.

Table A2. Baseline composition of clinical isolates in Phase 2.

	Germany	South Africa	TOTAL
**RIF-R**	118	100	218
**INH-R**	149	105	254
**MDR**	118	64	182
**Total MTB sputa**	**270**	**229**	**499**

Phase 2 sputum specimen selection

Sputum specimens tested at the Germany site came from Moldova and Azerbaijan and sputum specimens tested at the South Africa site were collected locally. Patients were selected due to their risk of drug resistance based on the high risk of MDR-TB in the geographic areas from which patients were sampled rather than patient-specific risk factors. Sputum specimens were collected consecutively at each of the three national reference laboratories and both smear positive and smear negative specimens were included. Patients on treatment were not excluded. Exclusion criteria were an insufficient volume of sputum or blood in the sputum.

Appendix B: Details of sequencing

The genetic characterisation of the strains was performed by Sanger DNA sequencing of previously identified regions involved in the development of resistance to the first-line drugs, i.e. *rpo*B (codons 209–694) for RIF and *kat*G (codons 268–328) plus *inh*A (position −148 to +60) for INH. Sequencing of the *rpo*B region of interest was performed according to the technique described by Telenti et al. (19), using primers TR8 GTGCACGTCGCGGACCTCCA, which is inclusive of codon 551 and TR9 TCGCCGCGATCAAGGAGT, which is inclusive of codon 499. Primers *inh*A 3F and *inh*A 4R (3) flanking the region encoding amino acid Ser94 of *inh*A were used to amplify a 517-bp *inh*A fragment. The primers *kat*G 290F (5'-ACT ACG GGC CGC TGT TTA TC-3') and *kat*G 583R (5'-TCCTTGCCCCAATAGACCTC -3') designed in this study were used to amplify a 250-bp fragment of *kat*G, which included the *kat*G region, codons 108-138 which is associated with resistance to INH. Direct sequencing of PCR products was carried out on an ABI Prism 3100 capillary sequencer (Applied Biosystems, CA, USA) and the ABI Prism BigDye Terminator kit v.1.1 according to the manufacturer’s instructions.

Appendix C: Characterization of indeterminate results

Table C1. Phase 1 Indeterminate results on initial and repeat testing of strains.

	RIF			INH		
**PHASE 1**	**HV1**	**HV2**	**Nipro**	**HV1**	**HV2**	**Nipro**
**Germany (initial testing)**	1/188 (0.5%)	1/188 (0.5%)	10/188 (5.3%)	1/188 (0.5%)	1/188 (0.5%)	8/188 (4.3%)
**Germany (after repeat testing)**	1/188 (0.5%)	1/188 (0.5%)	2/188 (1.1%)	1/188 (0.5%)	1/188 (0.5%)	1/188 (0.5%)
**South Africa (initial testing)**	1/190 (0.5%)	1/190 (0.5%)	2/190 (1.1%)	1/190 (0.5%)	1/190 (0.53%)	1/190 (0.53%)

Note: No repeat testing was performed in South Africa

Table C2. Phase 2 Indeterminate results for RIF and INH on initial testing.

		RIF-indeterminate % (n)			INH- indeterminate % (n)		
		**HV1**	**HV2**	**Nipro**	**HV1**	**HV2**	**Nipro**
**Overall** (n=474)		1.9% (9)	1.3% (6)	6.1% (29)	3.4% (16)	1.3% (6)	5.9% (28)
**By Site**	**Germany** (n=268)	1.1% (3)	0.4% (1)	6.3% (17)	1.1% (3)	0.8% (2)	6.0% (16)
	**South Africa** (n=206)	2.9% (6)	2.4% (5)	5.8% (12)	6.3% (13)	1.9% (4)	5.8% (12)
**By Smear-status**	**Positive** (n=449)	1.3% (6)	1.1% (5)	4.2% (19)	2.9% (13)	0.7% (3)	4.0% (18)
	**Negative** (n=25)	12.0% (3)	4.0% (1)	40.0% (10)	12.0% (3)	12.0% (3)	46.2% (10)
**By smear-grade**	**3+** (n=194)	1.0% (2)	1.0% (2)	2.6% (5)	2.1% (4)	0.0% (0)	2.6% (5)
	**2+** (n=106)	1.9% (2)	0.9% (1)	1.9% (2)	3.8% (4)	0.9% (1)	1.9% (2)
	**1+** (n=116)	0.9% (1)	1.7% (2)	5.2% (6)	2.6% (3)	1.7% (2)	4.3% (5)
	**Scanty** (n=33)	3.0% (1)	0.0% (0)	18.2% (6)	6.1% (2)	0.0% (0)	18.2% (6)

Note: Excludes contaminated cultures, culture-negatives and samples with testing performed was incomplete (e.g. missing results on one or several of the three LPAs)

Appendix D Details of reclassified strains according to sequencing.

Note that the ability of Sanger sequencing to pick up mixed infections is limited compared to that that of LPAs (resistant strain usually needs to make up >30% for sequencing while LPA can detect as low as 5-10%). This could lead to seemingly false-positive LPA results, i.e. it is possible that drug-sensitive results on sequencing and phenotypic DST are incorrect and that the LPA result is correct in such instances i.e. where there is heteroresistance and a disputed mutation.

Table D1. Reclassified strains according to sequencing for the determination of RIF resistance.

Strain	Species	RIF pDST	RIF cDST	Hain V1	Hain V2	Nipro	RIF: rpoB: nucleotide	RIF: rpoB: amino acid	Reason for reclassification
**G-041**	MTB	S	R	I	I	I	CTG 533 CCG	Leu 533 Pro	Disputed mutation within rpoB hotspot that confers resistance
**G-071**	MTB	S	R	R	R	R	CTG 533 CCG	Leu 533 Pro	Disputed mutation within rpoB hotspot that confers resistance
**SA-019**	MTB	S	R	S	S	S	TCG 531 TTG	Ser 531 Leu	Mutation within rpoB hotspot that confers resistance
**SA-069**	MTB	S	R	S	S	R	CTG 533 CCG	Leu 533 Pro	Disputed mutation within rpoB hotspot that confers resistance

pDST=phenotypic DST; cDST= composite reference standard DST; R = resistant, S = sensitive, I = indeterminate

Table D2. Reclassified strains according to sequencing for the determination of INH resistance.

Strain	Species	INH pDST	INH cDST	Hain V1	Hain V2	Nipro	INH katG amino acid	INH inhA nucleotide	Reason for reclassification
**G-014**	MTB	S	R	S	S	S	wild type	WT + C-15T	Mixed infection but C-15T confers resistance
**SA-037**	MTB	S	R	R	R	R	wild type	WT + C-15T	Mixed infection but C-15T confers resistance

pDST=phenotypic DST; cDST= composite reference standard DST; R = resistant, S = sensitive, I = indeterminate

Appendix E: Analysis of reasons of test failure (failure by design or failure by assay)

Appendix E illustrates an analysis of the reasons for test failure, broadly dividing these into failures by design and failures by assay.

Of the 6 strains for which there was LPA failure by design for RIF resistance detection (Table E1), 3 were due to other mutations outside of the hotspot targeted by the LPA, 2 were wild type strains likely to have an undetected mutation outside of the targeted hotspot and 1 had a rare mutation known to be associated with resistance outside of the hotspot.

Of the 11 strains for which there was LPA failure by assay for RIF resistance detection (Table E2), 6 were due to the presence of the Leu 533 Pro mutation: 4 of which were correctly identified as resistant by Nipro unlike Hain V1 and V2 which incorrectly identified all 6 of these as sensitive, 1 had another mutation in the hotspot that the assays should have detected, 3 were wild-type strains and 1 strain had a mixed resistance pattern.

Of the 12 strains for which there was LPA failure by design for INH resistance detection (Table E3), all were likely due to the presence of an undetected mutation outside of the targeted hotspot.

Of the 5 strains for which there was LPA failure by assay for INH resistance detection (Table E4), 3 were due to the presence of the C-15T mutation that should have been detected by the LPA (one of which strains demonstrated mixed resistance), 1 was due to the S315T mutation that should have also been detected by the LPA and 1 was a wild type strain not detected as such by the LPA.

Table E1. Failure by Design for RIF resistance detection.

Test strain	RIF pDST	RIF cDST	HV1	HV2	Nipro	RIF: rpoB: nucleotide	RIF: rpoB: amino acid	Reason for assay failure*
G-126 / SA-124	R	R	S	S	S	GTC 251 TTC	Val 251 Phe	Not a confirmed mutation associated with resistance; likely other mutation outside of known hotspot present that confers resistance
G-144 / SA-144	R	R	S	S	S	ATC 572 TTC	Ile 572 Phe	Rare mutation associated with resistance but outside of hotspot.
G-148 / SA-146	R	R	S	S	S	GTC 251 TTC	Val 251 Phe	Not a confirmed mutation associated with resistance; likely other mutation present outside of known hotspot that confers resistance
SA-049	R	R	S	S	S	wild type	wild type	Likely other mutation present outside of known hotspot that confers resistance
SA-052	R	R	S	S	S	569 GTC	569 Val	Not a confirmed mutation associated with resistance; likely other mutation present outside of known hotspot that confers resistance
SA-110	R	R	S	S	S	wild type	wild type	Likely other mutation present outside of known hotspot that confers resistance

pDST=phenotypic DST; cDST= composite reference standard DST; R = resistant; S= susceptible; SA= strain tested in South Africa; G = strain tested in Germany

* analysis performed based on review of the literature

Table E2. Failure by Assay for RIF resistance detection.

Test strain	pDST	cDST	HV1	HV2	Nipro	RIF: rpoB: nucleotide	RIF: rpoB: amino acid	Reason for assay failure
G-182	S	S	R	R	R	wild type	wild type	Wild type but not detected as such by LPAs
SA-018	S	S	R	R	R	wild type	wild type	Wild type but not detected as such by LPAs
SA-019	**S**	**R**	S	S	S	TCG 531 TTG	Ser 531 Leu	S531L is in rpoB hotspot and confers resistance
SA-057	R	R	S	S	R	CTG 533 CCG	Leu 533 Pro	L533P is in rpoB hotspot and confers resistance; Nipro gave correct result *
SA-060	R	R	S	S	S	mix WT + TCG 531 TTG	Mix: WT + Ser 531Leu	Mixed resistance but assays should have detected S531L, which is in rpoB hotspot and confers resistance *
SA-069	**S**	**R**	S	S	R	CTG 533 CCG	Leu 533 Pro	L533P is in rpoB hotspot and confers resistance; Nipro gave correct result
SA-076	R	R	S	S	S	CTG 533 CCG	Leu 533 Pro	L533P is in rpoB hotspot and confers resistance *
SA-090	R	R	S	S	S	CTG 533 CCG	Leu 533 Pro	L533P is in rpoB hotspot and confers resistance *
SA-104	R	R	S	S	R	CTG 533 CCG	Leu 533 Pro	L533P is in rpoB hotspot and confers resistance; Nipro gave correct result *
SA-181	S	S	R	R	R	wild type	wild type	Wild type but not detected as such by LPAs
SA-200	R	R	S	S	R	CTG 533 CCG	Leu 533 Pro	L533P is in rpoB hotspot and confers resistance Nipro gave correct result *

pDST=phenotypic DST; cDST= composite reference standard DST; R = resistant; S= susceptible; SA= strain tested in South Africa; G = strain tested in Germany

*Of note, the interpretation of the rpoB WT8 band for the Hain LPAs differed between sites. In Germany, a faint WT8 band was interpreted as resistant whereas this was interpreted as sensitive in South Africa, which may have given rise to some of the discordant results between phenotypic DST and LPA where the S531L and L533P mutations were involved. This suggests a faint band should be interpreted as resistant as this leads to better concordance with phenotypic DST.

Table E3. Failure by Design for INH resistance detection.

Test strain	pDST	cDST	HV1	HV2	Nipro	INH: katG: nucleotide	INH: katG amino acid	INH:inhA nucleotide	Reason for assay failure
G-070 / SA-068	R	R	S	S	S	wild type	wild type	wild type	Likely other mutation present outside of known hotspot that confers resistance
G-073 / SA-071	R	R	S	S	S	wild type	wild type	wild type	Likely other mutation present outside of known hotspot that confers resistance
G-091 / SA-087	R	R	S	S	S	wild type	wild type	wild type	Likely other mutation present outside of known hotspot that confers resistance
G-116 / SA-115	R	R	S	S	R*	ACC 324 CCC	Thr 324 Pro	wild type	ACC324CCC located in hotspot but not known to be associated with resistance; Likely other undetected mutation outside of hotspot that confers resistance
G-134 / SA-131	R	R	S	S	S	wild type	wild type	wild type	Likely other mutation present outside of known hotspot that confers resistance
G-205	R	R	S	S	S	wild type	wild type	Not tested	Likely other mutation present outside of known hotspot that confers resistance
SA-048	R	R	S	S	S	wild type	wild type	wild type	Likely other mutation present outside of known hotspot that confers resistance
SA-049	R	R	S	S	S	wild type	wild type	wild type	Likely other mutation present outside of known hotspot that confers resistance
SA-053	R	R	S	S	S	wild type	wild type	wild type	Likely other mutation present outside of known hotspot that confers resistance
SA-103	R	R	S	S	S	wild type	wild type	wild type	Likely other mutation present outside of known hotspot that confers resistance
SA-127	R	R	S	S	S	wild type	wild type	wild type	Likely other mutation present outside of known hotspot that confers resistance
SA-196	R	R	S	S	S	wild type	wild type	wild type	Likely other mutation present outside of known hotspot that confers resistance
SA-204	R	R	S	S	S	Not tested	Not tested	Not tested	N/A

pDST=phenotypic DST; cDST= composite reference standard DST; R = resistant; S= susceptible; SA= strain tested in South Africa; G = strain tested in Germany. *It is unclear why the S9 probe for Nipro picked up the Thr324Pro mutation.

Table E4. Failure by Assay for INH resistance detection.

Test strain	pDST	cDST	HV1	HV2	Nipro	INH katG: nucleotide	INH katG amino acid	INH inhA: nucleotide	Reason for assay failure
G-014	S	R	S	S	S	wild type	wild type	WT + C-15T	Mixed resistance but assays should have detected C15T
G-118/ SA-116	R	R	S	S	S	wild type	wild type	C -15 T	Assay should have detected C15T as R
SA-052	R	R	S	S	R	AGC 315 ACC	Ser 315 Thr	G-47C	Hain V1 and V2 should have detected S315T as R (as Nipro did); G-47C not associated with resistance
SA-112	R	R	R	R	S	wild type	wild type	C -15 T	Nipro should have detected C15T as R as Hain V1 and V2 did
SA-184	S	S	R	R	S	wild type	wild type	wild type	Wild type strain but not detected as such by Hain V1 and V2

pDST=phenotypic DST; cDST= composite reference standard DST; R = resistant; S= susceptible; SA= strain tested in South Africa; G = strain tested in Germany

Appendix F.

Ease-of-use questionnaire results

Since all three LPAs are based on the same technique, it was hypothesized that there would be no major difference in the technical complexity required to perform each assay. However the ease-of-use questionnaire (detailed below) revealed that Hain V2 was easier to perform than Hain V1 due to the modifications in DNA amplification and extraction steps, as well as easier to interpret. Overall ease-of-use was also good for Nipro compared to Hain V1, with some operators reporting clearer bands that were easier to interpret, although others found that the smaller strips more difficult to process. In summary, ease-of-use was considered good for all tests. Operators rated them between extremely easy to fairly easy to use.

	Genotype MTBDRplus V1	*Genotype MTBDRplus V2*	*Nipro NTM+MDRTB*
**Based on your experience with each device, how would you grade ease of use?**	Extremely easy (1/4)<br>Fairly easy (2/4)<br>Neutral (1/4)<br>Fairly difficult<br>Extremely difficult	Extremely easy (1/4)<br>Fairly easy (3/4)<br>Neutral<br>Fairly difficult<br>Extremely difficult	Extremely easy<br>Fairly easy (4/4)<br>Neutral<br>Fairly difficult<br>Extremely difficult
**Do you find it easy to discriminate between positive and negative bands when looking at the strips?**	Extremely easy (1/4)<br>Fairly easy (3/4)<br>Neutral<br>Fairly difficult<br>Extremely difficult	Extremely easy (1/4)<br>Fairly easy (3/4)<br>Neutral<br>Fairly difficult<br>Extremely difficult	Extremely easy (2/4)<br>Fairly easy<br>Neutral (2/4)<br>Fairly difficult<br>Extremely difficult
**Do you find it easy to interpret whether a test is Resistant or Sensitive when looking at the strips?**	Extremely easy (2/4)<br>Fairly easy (2/4)<br>Neutral<br>Fairly difficult<br>Extremely difficult	Extremely easy (3/4)<br>Fairly easy (1/4)<br>Neutral<br>Fairly difficult<br>Extremely difficult	Extremely easy (2/4)<br>Fairly easy<br>Neutral (2/4)<br>Fairly difficult<br>Extremely difficult

**How would you judge the complexity of the assay compared with Hain v.1?**	NA	More complex<br>Same (1/4)<br>Less complex (3/4)	More complex (2/4)<br>Same (2/4)<br>Less complex
